# A retrospective analysis of the efficacy of baseball suture method in single-port laparoscopic myomectomy

**DOI:** 10.12669/pjms.41.8.10941

**Published:** 2025-08

**Authors:** Kang Wu, Dandan Shen

**Affiliations:** 1 Kang Wu Department of Gynecology, Jiaxing Maternity and Child Health Care Hospital, College of Medicine, Jiaxing University, Jiaxing 314000, China; 2 Dandan Shen Department of Gynecology, Jiaxing Maternity and Child Health Care Hospital, College of Medicine, Jiaxing University, Jiaxing 314000, China

**Keywords:** Baseball Suture, Bleeding, Gynecological Surgery, Hemostasis, Myomectomy, Single-port Laparoscopy

## Abstract

**Objective:**

To assess the hemostatic effect of baseball suture after myomectomy under single-port laparoscopy.

**Methodology:**

Retrospective review of 91 patients admitted to Department of Gynecology, Jiaxing Maternity and Child Health Care Hospital from July 2021 to August 2023 who had myomas removed via single-port laparoscopy. We counted the suture method (traditional suture method vs baseball suture method) of each case divided into two groups (41 cases in control group; 50 cases in experimental group), and assessed the amount of intraoperative bleeding, postoperative hemoglobin decrease, and pelvic effusion. Propensity score matching (PSM) between groups was performed by the maximum diameter of the myoma, patient’s age, number of myomas, type of myoma, and location of myoma.

**Results:**

After PSM, a total of 38 pairs were matched. The mean amount of intraoperative bleeding, postoperative hemoglobin decrease, and pelvic fluid accumulation in the control group were significantly higher than those in the experimental group (115.79ml vs 94.34ml; 16.55g/l vs 12.79g/l; 146.74ml vs 119.13ml P<0.05). There was no significant difference in operation time between the two groups (83.84min vs 87.79min P>0.05).

**Conclusions:**

Our data suggested that using baseball suture method can significantly reduce the amount of intraoperative bleeding, postoperative hemoglobin decrease, and pelvic fluid accumulation in patients undergoing single-port laparoscopic myomectomy compared with traditional suture method. In addition, although using baseball suture method is more cumbersome, it does not significantly increase operation time.

## INTRODUCTION

**Fig.1:**
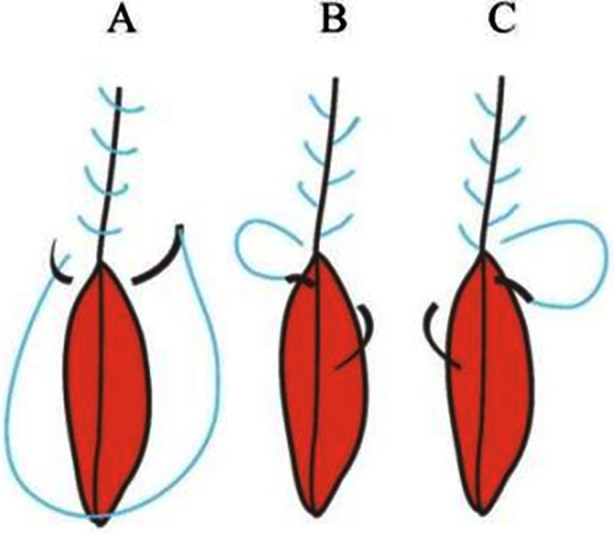
Schematic diagram of suturing. A: Traditional suture; B: The first needle of baseball suture; C: The second needle of baseball suture.

Uterine myomas are the most common benign tumors among women of childbearing age, with a prevalence rate reaching up to 80% across different ethnicities and countries.^1^-^4^ For symptomatic patients wishing to preserve fertility, surgical removal(myomectomy) is the preferred treatment.^5^ In recent years, single-port laparoscopic surgery has gained traction in gynecological practice, especially in China, due to its smaller incisions, superior cosmetic outcomes, and faster postoperative recovery.^6^ Nevertheless, the limited operative space in single-port laparoscopy poses challenges in achieving effective hemostasis and managing postoperative complications such as bleeding and pelvic fluid accumulation.^7^ Surgeons in our department select suturing techniques based on personal preference, with results from different methods being similar. Recently, baseball suturing has gained popularity due to its potential for better hemostasis, despite being more complex^8^-^9^ (Fig.1). This study retrospectively analyzed the outcomes of different suturing methods in single-port laparoscopic myomectomy, comparing their effects on intraoperative conditions and postoperative recovery.

## METHODOLOGY

We retrospectively analyzed 91 cases of single-port laparoscopic myomectomy (Type-II submucous and intramural uterine myomas) performed in Department of Gynecology, Jiaxing Maternity and Child Health Care Hospital between July 2021 and August 2023. All procedures followed standardized protocols and were conducted by multiple surgeons. Of these cases, 50 underwent baseball suturing (experimental group) and 41 used traditional suturing (control group).

### Ethical approval:

All procedures performed in studies involving human participants were in accordance with the ethical standards of the institutional and/or national research committee and with the 1964 Helsinki declaration and its later amendments or comparable ethical standards. The study was approved by the ethics committee of Jiaxing Maternity and Child Health Care Hospital with the number 2024-Y-80 on October 22th, 2024.

Both groups underwent surgery under general anesthesia with endotracheal intubation. The control group received traditional suturing with Stratafix (Angiotech, USA) for the myometrium and serosa layers, while the experimental group used baseball suturing with barbed absorbable sutures. In both groups, a midline umbilical incision (1.5–2 cm) was made using a Kang Ji disposable single-port cannula (Kang Ji, Hangzhou, China). Oxytocin (40U, Minsheng, Hangzhou, China) was administered postoperatively via intravenous infusion to promote uterine contraction, and cefuroxime was used for infection prevention. For patients allergic to cephalosporins, clindamycin was administered.

Key surgical indicators, including operation time and intraoperative blood loss, as well as postoperative indicators like hemoglobin decline and pelvic fluid volume, were compared between the two groups. Intraoperative blood loss was calculated by subtracting the volume of saline used for flushing from the total fluid aspirated from the abdominal cavity. Surgery duration was measured from skin incision to closure. Hemoglobin decrease was assessed via complete blood count, and pelvic fluid volume was measured by ultrasound.

### Statistical Analysis:

The data was processed using SPSS version 26 statistical software (IBM Corp, Armonk, NY, USA). In the case of normally distributed measurement data, it was presented in the form of (x±s), and the independent sample t-test was employed for inter-group comparison. For skewed distributed measurement data, it was expressed as M (P25, P75), with the Mann-Whitney U test being utilized for comparison between groups. Count data was represented as rates or constituent ratios, and the χ² test or Fisher’s exact test was adopted for inter-group comparison. A P value of less than 0.05 signified a statistically significant difference. A 1:1 propensity score matching (PSM) analysis was carried out to mitigate the impact of confounding factors and to adjust the disparities between the experimental group and the control group. The PSM was constructed via a logistic regression model. Based on the PSM similarity, the closest available matches between the two groups were selected. The matching tolerance was set at 0.02.

## RESULTS

Preliminary statistics showed that patients in the experimental group were aged 27–52 years (mean age: 39.32±6.93 years), with a maximum myoma diameter of 6.61±1.26 cm. This group included four cases with two myomas, four cases with Type-II submucous myomas, and nine cases with myomas located on the uterine posterior wall. In the control group, patients were aged 26–54 years (mean age: 39.71±6.57 years), with a maximum myoma diameter of 6.76±1.19 cm. This group included five cases with two myomas, four with Type-II submucous myomas, and eight with posterior wall myomas. After 1:1 propensity score matching (PSM) based on age, maximum myoma diameter, number of myomas, myoma location, and type, 38 matched pairs were selected, and no significant differences were found between the groups (P>0.05), ensuring data comparability.

During the operation, the total operation time and intraoperative blood loss were recorded, and then statistical analysis was performed (Table-I). It was found that there was no significant difference in total operation time between the two groups caused by the two different suturing methods (P>0.05). There was a significant difference in intraoperative blood loss between the two groups, which was statistically significant (P<0.05).

**Table-I:** Comparison of patient-related information and surgery-related indicators.

Variables	Overall			Matched pairs by PSM		
	TS (n=41)	BS (n=50)	P value	TS (n=38)	BS (n=38)	P value
Patient age, years	39.70±6.57	39.32±6.93	0.787	39.03±6.21	39.08±7.14	0.973
Diameter of largest myoma, cm	6.76±1.19	6.61±1.26	0.567	6.64±1.09	6.64±1.09	0.559
***Number of enucleated myoma***						
1	36	46	0.726	33	35	0.711
2	4	4		5	3	
***Type of myoma myoma***						
Type 2 submucous myoma	4	4	0.768	3	3	1
intramural myoma	37	46		35	35	
***Location of largest myoma***						
Anterior wall	33	41	0.854	31	30	0.773
Posterior wall	8	9		7	8	
Surgical duration, min	84.41±11.32	88.1±11.21	0.124	83.84±11.02	87.79±12.23	0.144
Total blood loss, mL	120.37±53.41	90.60±40.22	0.003	115.79±47.75	94.34±41.86	0.041
postoperative hemoglobin decrease, g/L	16.87±5.27	12.38±4.83	<0.001	16.55±5.02	12.79±4.81	0.001
pelvic fluid accumulation, mL	149.90±62.43	115.54±53.5	0.006	146.74±63.55	119.13±56.37	0.049

Data are expressed by median. TS, traditional suture; BS, baseball suture; PSM, propensity score matching.

After surgery, the recovery conditions of the two groups of patients were recorded and analyzed (Table-I). The difference in the decrease in hemoglobin between the two groups of patients after surgery was statistically significant (P<0.05). The difference in the amount of pelvic fluid between the two groups of patients after surgery was also statistically significant (P<0.05).

## DISCUSSION

This study retrospectively analyzed the hemostatic effects of traditional suturing compared with the baseball suture method in single-port laparoscopic myomectomy. By comparing the surgical outcomes of the two groups, we found that the baseball suture method significantly reduced intraoperative blood loss, postoperative hemoglobin decrease, and pelvic fluid accumulation, with no significant difference in operation time. These findings indicate that the baseball suture method provides superior hemostasis without extending surgical duration. Uterine myomas are the most common benign tumors among women of reproductive age.^1^-^3^ When they enlarge and are accompanied by symptoms such as abnormal uterine bleeding, anemia, or compression, myomectomy remains the preferred treatment option, particularly for patients desiring to preserve fertility.^10^-^13^ Traditional multi-port laparoscopic myomectomy remains widely adopted globally.^14^ However, in recent years, single-port laparoscopic surgery has gained traction in gynecological practice, especially in China, due to its smaller incisions, superior cosmetic outcomes, and faster postoperative recovery.^15^ Nevertheless, the limited operative space in single-port laparoscopy poses challenges in achieving effective hemostasis and managing postoperative complications such as bleeding and pelvic fluid accumulation. Various techniques have been explored to optimize single-port laparoscopic myomectomy, including the use of barbed sutures^16^ and medications such as oxytocin and pituitrin.^17^

The baseball suture method has been widely recognized for its effectiveness in achieving hemostasis in multi-port laparoscopic surgery.^18^ The baseball suture technique involves inserting the needle from the inner base of the myoma cavity and exiting through the serosal layer of the uterine wall at the incision edge. This technique achieves continuous full-thickness suturing, providing uniform pressure to prevent needle-hole bleeding and reducing the risk of myometrial tearing.^19^,^20^

Despite its proven efficacy, the application of the baseball suture in single-port laparoscopic surgery, where operative space is more restricted, is still under exploration. Our study demonstrated that the baseball suture method yields better hemostatic outcomes in single-port laparoscopic myomectomy than traditional suturing. Consistent with previous research, this technique significantly reduced postoperative hemoglobin decline and the need for blood transfusion. Some studies have noted that the baseball suture method may increase surgical duration due to its complexity, but this appears to be highly dependent on the surgeon’s skill and experience.^21^

A key strength of our study lies in the use of 1:1 propensity score matching (PSM), which controlled for confounding factors such as patient age, myoma size, number, type, and location. This method ensured greater comparability between groups and allowed us to draw more robust conclusions. The findings reinforce the baseball suture method’s advantages in reducing intraoperative bleeding, postoperative anemia, and pelvic effusion, highlighting its potential clinical significance, especially in preserving fertility and enhancing recovery. Although the baseball suture method requires advanced surgical skills, our data showed that it does not significantly prolong operation time. Skilled surgeons can perform the technique efficiently, leveraging its hemostatic benefits without adding considerable time to the procedure. While technically demanding, the substantial reduction in postoperative bleeding and complications underscores its value in clinical practice, particularly for cases requiring meticulous hemostasis.

### Limitations:

First, the relatively small sample size-91 cases, with 38 pairs matched through PSM-may limit the generalizability of our findings. Future studies with larger, multi-center prospective cohorts are needed to validate these results. Second, individual patient factors, such as underlying health conditions and variations in surgical proficiency, might have influenced outcomes. Although efforts were made to control these variables, their complete elimination was not feasible. Future research should aim to further address these external factors. Additionally, this study focused primarily on short-term outcomes such as intraoperative blood loss and immediate postoperative recovery. Future investigations could evaluate long-term outcomes, including uterine function, pelvic adhesion rates, and fertility. Such research would provide a more comprehensive assessment of the baseball suture method’s clinical utility.

In conclusion, the baseball suture method demonstrated superior hemostatic efficacy compared to traditional suturing techniques in single-port laparoscopic myomectomy, significantly reducing intraoperative bleeding, postoperative anemia, and pelvic fluid accumulation without prolonging operation time. Despite its technical complexity, its substantial clinical benefits make it a valuable technique in gynecological surgery. Future large-scale studies are necessary to confirm its long-term outcomes and broaden its applicability, thereby promoting its widespread adoption in clinical practice.

## CONCLUSION

Our data suggest that the use of baseball sutures for wound closure during single-port laparoscopic myomectomy is effective in reducing intraoperative patient bleeding, postoperative hemoglobin drop, and pelvic effusion. In addition, the use of baseball sutures did not significantly increase the operative time, so we believe that the use of baseball sutures in the clinic will bring more benefits to the patients. More clinical data are needed to validate the feasibility of its wider application.

### Author’s Contributions:

**KW:** Literature search, Conceived and designed the study.

**KW and DS:** Collected the data and performed the analysis, prepared the tables. Critical review.

**KW:** Writing of the manuscript and is responsible for the integrity of the study.

All authors have read and approved the final manuscript.
